# Editorial for the Special Issue “A Themed Issue in Honor of Professor Hartmut Derendorf—Outstanding Contributions in the Fields of Quantitative Clinical Pharmacology”

**DOI:** 10.3390/antibiotics12020353

**Published:** 2023-02-08

**Authors:** Françoise Van Bambeke, Sebastian Wicha, Paul M. Tulkens, Markus Zeitlinger

**Affiliations:** 1 Pharmacologie Cellulaire et Moléculaire, Louvain Drug Research Institute, Université catholique de Louvain, 1200 Brussels, Belgium; 2 Department of Clinical Pharmacy, Institute of Pharmacy, University of Hamburg, 20146 Hamburg, Germany; 3 Department of Clinical Pharmacology, Medical University of Vienna, 1090 Vienna, Austria

Pharmacokinetics (PK) is the discipline investigating the absorption, distribution, metabolization and elimination of a drug in the body. This term was first introduced in 1953 by Prof Hartmut Dost, a pediatrician and active member of the German Pharmacological Society, who was concerned with the possibility of adapting dose regimens in children based on adult dosages [1].

Pharmacodynamics (PD), on the other side, studies the biochemical, molecular and physiological effects of drugs. The origin of this term is less described, but it possibly emerged in the middle of the 19th century. Soon later, in 1888, the German Oswald Schmiedeberg published a book, *Fundamentals of Drugs*, for which he is considered as the father of modern pharmacology [2].

We thus see that German scientists have been at the forefront of these disciplines. Hartmut Derendorf, to whom this Special Issue is dedicated, belongs to this dynasty of great German pharmacologists. One of his first papers, indeed, was dedicated to the PK/PD of analgesics [3], i.e., determining the link between drug exposure and its effect, which is ultimately what is important for clinical practice. Early on, he also claimed the importance of determining the unbound concentrations of drugs, and of antibiotics in particular, at their site of action and developed microdialysis protocols for this purpose [4].

In the field of antibiotic pharmacology, PK/PD concepts started to become popular in the early 1990s, with the definition of drugs for which efficacy was related to T > MIC, AUC/MIC or peak/MIC, thanks to the visionary mind of William A. Craig [5]. H. Derendorf jumped into this discipline, working in parallel on pain-relieving medicines and anti-infective agents. In his turn, he brought a huge building block to the construction of modern pharmacology by suggesting the idea of modeling PK/PD relationships in order to predict the effects based on the concentrations [6]. From these early premises emerged pharmacometrics (or quantitative pharmacology). This modern discipline proposes mathematical models to describe the relationship between exposure to drugs (PK) and their effects on the body or the evolution of the disease (PD). At the turn of the century, the FDA recommended the use of PK/PD modeling in order to define the appropriate dosing for new drugs and to include these data in the new drug application forms [7], while the question was still under debate by the EMA at that time [8].

Over the forty years of the active career of H. Derendorf, science has markedly evolved, to the benefit of patient care, moving from the basic knowledge of drug action and fate in the body, to the recent recognition of the necessity of dosing individualization in critically ill patients, which has been made possible thanks to the development of tools to which H. Derendorf significantly contributed. During his entire career, H. Derendorf did not hesitate to challenge the shortcomings of established paradigms such as the MIC method as a gold standard for describing the antimicrobial activity of a certain antibiotic–bug combination. As a testimony to his heritage, his last contribution was a co-authorship in a position paper entitled “Research priorities towards precision antibiotic therapy to improve patient care” [9].

In this Special Issue, 11 groups from all over the world have brought specific contributions to the fields of antibiotic pharmacokinetics, pharmacodynamics and pharmacometrics, to honor the outstanding contributions of H. Derendorf in the field of quantitative clinical pharmacology.
